# Are Home Care Aides Interested in Helping Clients Do Physical Activity?

**DOI:** 10.1093/geroni/igaa057.1432

**Published:** 2020-12-16

**Authors:** Naoko Muramatsu, Lijuan Yin, Maria Caceres

**Affiliations:** University of Illinois at Chicago, Chicago, Illinois, United States

## Abstract

The current home care paradigm dictates home care aides (HCAs) provide prescribed help with activities of daily living, rather than stimulating older adults’ reserves to maintain independence. Little is known about whether HCAs are interested in expanding their role to promote their clients’ health. This study examined HCAs’ interest in helping clients do physical activity among workers who care for their family members or clients assigned by home care agencies in a Medicaid-funded home care program. Data came from brief surveys completed by HCAs at state-mandated in-service training sessions (N=602; 42% caring for non-family clients only, 38% family clients only, 20% both family and non-family clients). Ordered logit analysis was conducted to examine whether HCAs caring for family clients differ from those caring for non-family clients in levels of interest in helping clients do safe physical activity as part of home care work, controlling for HCAs’ age, gender, language (English/Spanish), and years of home care experience. HCAs’ interest levels were high (62% very, 21% somewhat, 8% slightly, 9% not at all interested). Ordered logit analysis indicated that HCAs caring for family members had significantly higher interest levels than those caring for non-family clients only (83% higher among HCAs caring for both family and non-family clients, 30% higher among HCAs caring for family clients only). To reflect HCAs’ interests and to maintain clients’ independence, home care workforce training should direct its attention to empower HCAs to assume health-promoting roles.

